# Familial platelet disorders with a predisposition to acute myelogenous leukaemia: a RUNX1 update

**DOI:** 10.1186/1897-4287-10-S2-A64

**Published:** 2012-04-12

**Authors:** J Rossini, B Mercorella, S Townshend, C Vakulin, L Rawlings, X Li, C Hahn, H Scott

**Affiliations:** 1 Institute of Medical and Veterinary Science, Adelaide, SA, Australia; 2 Centre for Cancer Biology, SA Pathology, Adelaide, SA, Australia; 3 Genetics Services of WA, KEMH, Subiaco, WA, Australia

## Background

Familial platelet disorder with a predisposition to acute myelogenous leukaemia (FPD-AML, omim#601399) is an autosomal dominant disorder that is linked to mutations within the RUNX1 gene. The RUNX1 gene, present on 21q22.1, plays a role as a regulatory switch in both embryonic and adult haemopoietic development. Heterozygous mutations in RUNX1 are a common feature in FPD-AML with different prognostic outcomes reported to be attributable to the location of the mutation within the protein domains.

The Australian Familial Haematological Cancer Study (AFHCS) currently has 56 Australian families registered with predispositions to haematological malignancy. RUNX1 mutations have been found in 11 patients from 3 families diagnosed with AML. This figure is predicted to be higher if screening occurred when FPD was first detected.

The IMVS, Adelaide, now offers full RUNX1 gene screening. To date, we have screened 25 individuals and confirmed germline mutations in 2 AFHCS families. We have reported a novel germline heterozygous nonsense mutation (c.958C>T, p.Arg320X), and a deletion of exons 2,3 and 4 (c.59-32857_508+2502del).

Recent research is highlighting the role of monoallelic RUNX1 mutations in the generation and progression of pre-leukaemic FPD to AML. The evidence suggests that different prognostic outcomes are dependent on the impact the mutation has on the final product, although there is a wide degree of genetic heterogeneity observed. This has implications for the management and treatment options available to individuals affected. It has also proved useful in selecting family members negative for the familial mutation who may be suitable as bone marrow donors.

